# The association between hypertension and other cardiovascular risk factors among non-diabetic Saudis adults–A cross sectional study

**DOI:** 10.1371/journal.pone.0246568

**Published:** 2021-02-23

**Authors:** Ghada M. A. Ajabnoor, Suhad Bahijri, Aliaa Amr Alamoudi, Rajaa Al Raddadi, Jawaher Al-Ahmadi, Hanan Jambi, Anwar Borai, Jaakko Toumilehto

**Affiliations:** 1 Department of Clinical Biochemistry, Faculty of Medicine, King Abdulaziz University, Jeddah, Saudi Arabia; 2 Saudi Diabetes Study Research Group, King Fahd Medical Research Center, King Abdulaziz University, Jeddah, Saudi Arabia; 3 Department of Community Medicine, Faculty of Medicine, King Abdulaziz University, Jeddah, Saudi Arabia; 4 Department of Family Medicine, Faculty of Medicine, King Abdulaziz University, Jeddah, Saudi Arabia; 5 Department of Food and Nutrition, Faculty of Human Sciences and Design, King Abdulaziz University, Jeddah, Saudi Arabia; 6 King Abdullah International Medical Research Center, King Saud Bin Abdulaziz University for Health Sciences, Pathology, King Abdulaziz Medical City, Jeddah, Saudi Arabia; 7 Public Health Solutions, Finnish Institute for Health and Welfare, Helsinki, Finland; 8 Department of Public Health, University of Helsinki, Helsinki, Finland; Government College University Faisalabad, Pakistan, PAKISTAN

## Abstract

Population specific associations between cardiovascular disease with various risk factors including pre-hypertension and hypertension were reported. We aimed to investigate the association of higher than optimal blood pressure with measures of dysglycemia, dyslipidemia, and markers of inflammation in non-diabetic Saudi adults hoping to improve current Saudi guidelines to prevent cardiovascular disease. Volunteers were recruited randomly from public healthcare centers in Jeddah. Demographic information, blood pressure (BP), and anthropometric measurements were taken. Fasting blood samples were drawn, then again following 1-hour oral glucose tolerance test. Glycated hemoglobin, fasting plasma glucose (FPG), lipid profile, highly sensitive C- reactive protein, gamma glutamyl transferase, and 1-hour plasma glucose were measured. Complete data was found for 742 men and 592 women. Pre-hypertension was found in 47.2% of men, and 24.7% of women, while 15.1% of men, and 14.6% of women were hypertensive. Means of measured variables differed significantly between normotensive, pre-hypertensive, and hypertensive groups of men and women in gender specific manner. Association between measured variables and elevated BP, and hypertension were assessed using logistic regression models. After adjustment for age, body mass index and waist circumference, elevated blood pressure was associated with elevated triglycerides in men, while hypertension was significantly associated with elevated fasting plasma glucose, total cholesterol, triglycerides, low density lipoprotein- cholesterol, and low high density lipoprotein- cholesterol in men, and elevated triglycerides, and total cholesterol in women. Therefore, it is strongly recommended to measure lipid profile, specifically TG, for all diagnosed pre-hypertensive and hypertensive patients in addition to FPG for men.

## Background

Type 2 diabetes (T2D) is a risk factor for cardiovascular diseases (CVD) [1]. Dyslipidemia, smoking, chronic inflammation, and abdominal obesity are also known risk factors for CVD [2–6]. Moreover, various epidemiological studies have indicated that blood pressure (both diastolic and systolic) is a most important risk factor, which has an independent, continuous, and direct association with CVD outcomes [7–10]. Furthermore, the increased risk of CVD was found to be associated with high-normal blood pressure (BP) values [11]. Obesity, hypertension, dyslipidemia and impaired glucose tolerance (IGT) had been found to cluster together [12–17], thus suggesting the possibility of a common aetiological mechanism, with the association between elevated BP and those other cardiovascular risk factors having long been acknowledged [18]. It has also been reported that obesity, and abdominal obesity are risk factors for the other CVD risk factors: hypertension, dyslipidemia, and diabetes mellitus [19]. Evidence for the relationship between increased weight and increased BP was reported much earlier based on findings in the nationwide "Community Hypertension Evaluation Clinic Screening" of more than 1 million men and women in the USA [20], and the "Framingham Study", where it was demonstrated that BP increased in both men and women with increased overweight [21]. These findings were verified later in a Canadian study especially in young adults 18 to 34 years of age [22], and in female nurses in USA [23].

Moreover, the presence of other cardiovascular risk factors, which cause endothelial dysfunction, has been suggested to contribute to the pathophysiology of hypertension [24], and studies of the association between hypertension and other CVD risk factors are numerous. A Japanese study showed that high normal BP, and hypertension increased the risk of T2D [25]. In addition, elevated BP was found to be increased in people with impaired glucose tolerance (IGT) in young, American, black adults [26]. Furthermore, a large meta-analysis of 4.1 million people showed that high BP was associated with elevated incidence of T2D [3]. Moreover, a more recent study reported that people with high BP are nearly 60% more likely to develop T2D [27], and a longitudinal Chinese study reported that time-cumulated exposure to elevated BP was significantly associated with an elevated incidence of IGT and diabetes [28]. Association between dyslipedemia and elevated BP has also been investigated, and is well discussed by Ruben et al (2006) [29]. Dyslipidemia leads to endothelial damage resulting in loss of vasomotor activity, which could cause hypertension (Nickenig et al, 2002) [30]. Similarly, elevated BP had been reported to be associated with inflammation in the general population [31, 32], and even at the typically observed low levels of inflammation in the general population, there is an association between the level of C-reactive protein (CRP), as a marker of inflammation, and systolic BP (SBP) [33].

Studies on mentioned associations did not include people from the Arab world. Genetic factors, and hence ethnicity play an important role in susceptibility to disease [34, 35], In an earlier study, conducted in Jeddah, Saudi Arabia, [36], we reported an association between above than normal weight with dysglycemia, and hypertension, which was gender specific. Moreover, in another survey, conducted in the same city at a later date, high LDL-C was found to be associated with an increased probability of pre-diabetes (Al amri et al 2019), which points to the clustering of the three CVD risk factors, excess weight increased blood pressure and dysglycemia in the Saudi population, as noted in studies from other countries [12–17]. Up to date, there are no official guidelines for laboratory investigations in non-diabetic people with hypertension in Saudi Arabia. Therefore, we aimed at investigating the association between higher than optimal blood pressure and other known laboratory measured risk factors for CVD, including measures of dysglycemia, lipid profile, C-reactive protein (CRP) [37, 38], and gamma glutamyl transferase (GGT) [39–41], in a non- diabetic Saudi population in the hope of providing evidence for improved management to prevent the progression to CVD.

## Materials and methods

### Study design and sample collection

Data included in this study were obtained from a survey conducted in the city of Jeddah between July 2016 and February 2017. Approval was given by the Committee on the Ethics of Human Research at the Faculty of Medicine-King Abdulaziz University, Jeddah (Reference No. 338–10). A full explanation of sampling methodology has been outlined earlier [42], and is summarized here as follows: non-diabetic adults (age ≥18 years) were recruited from attendees of Primary Health Care (PHC) centers in Jeddah, Saudi Arabia. Following signing an informed consent form, demographic, dietary, and lifestyle variables, as well as medical history and family history of chronic diseases were collected from recruits using a predesigned questionnaire. Fasting blood sample was taken, for determination of fasting plasma glucose (FPG), glycated hemoglobin (HbA1c), lipid profile, high-sensitivity CRP (hs-CRP) and GGT, followed by 1-hour oral glucose tolerance test (OGTT) [43, 44] to screen for diabetes and pre-diabetes. Anthropometric measurements [weight, height, waist circumference (WC)], and blood pressure (BP) were measured using standardized equipment and techniques. Weight and height were used to calculate the body mass index (BMI).

### Biochemical assays

Whole blood, serum and plasma samples were sent regularly to an accredited laboratory by the College of American Pathologist at the National Guard Hospital in Jeddah. Plasma glucose and serum hs-CRP, GGT, total cholesterol (TC), high density lipoprotein-cholesterol (HDL-C) and triglycerides (TG) levels were measured by spectrophotometric methods using Architect c8000 auto-analyzer (ABBOTT- USA). Low-density lipoprotein- cholesterol (LDL-C) was calculated using the Friedewald equation [45]. HbA1c was measured with high pressure liquid chromatography (HPLC) using automated HbA1c analyzer G8 (TOSOH Corporation-Japan).

#### a) Diagnosis of pre-hypertension and hypertension

Pre-hypertension was defined according to recommendations by the Joint National Committee on Prevention, Detection, Evaluation, and Treatment of High Blood Pressure (JNC) VII report [46] as systolic blood pressure: 120 to 139 mm Hg, and diastolic blood pressure 85 to 89 mm Hg, while hypertension was defined as SBP ≥140 and/or DBP ≥90 mm Hg or taking antihypertensive drug treatment.

### Statistical analysis

IBM SPSS statistics version 20.0 for Windows was used to enter and analyze collected data. The baseline characteristics of study population were calculated statistically and described as mean, standard deviations (SD), and frequencies.

Demographic, lifestyle and clinical factors of elevated blood pressure were analyzed by comparing participants who were identified to have pre-hypertension or hypertension and those with normal blood pressure. Factors with continuous variables were analyzed using ANOVA to compare the three groups, and independent t-test to compare two groups, while those with categorical variables were analyzed using Chi-square test or Fisher’s exact test, as appropriate. Fisher’s exact test was applied when the number of people per cell was 5 or less.

Unadjusted and adjusted logistic regression models were used for assessing association between laboratory measured risk factors for CVD and outcome variables: elevated BP, and hypertension.

## Results

**Fig 1:**
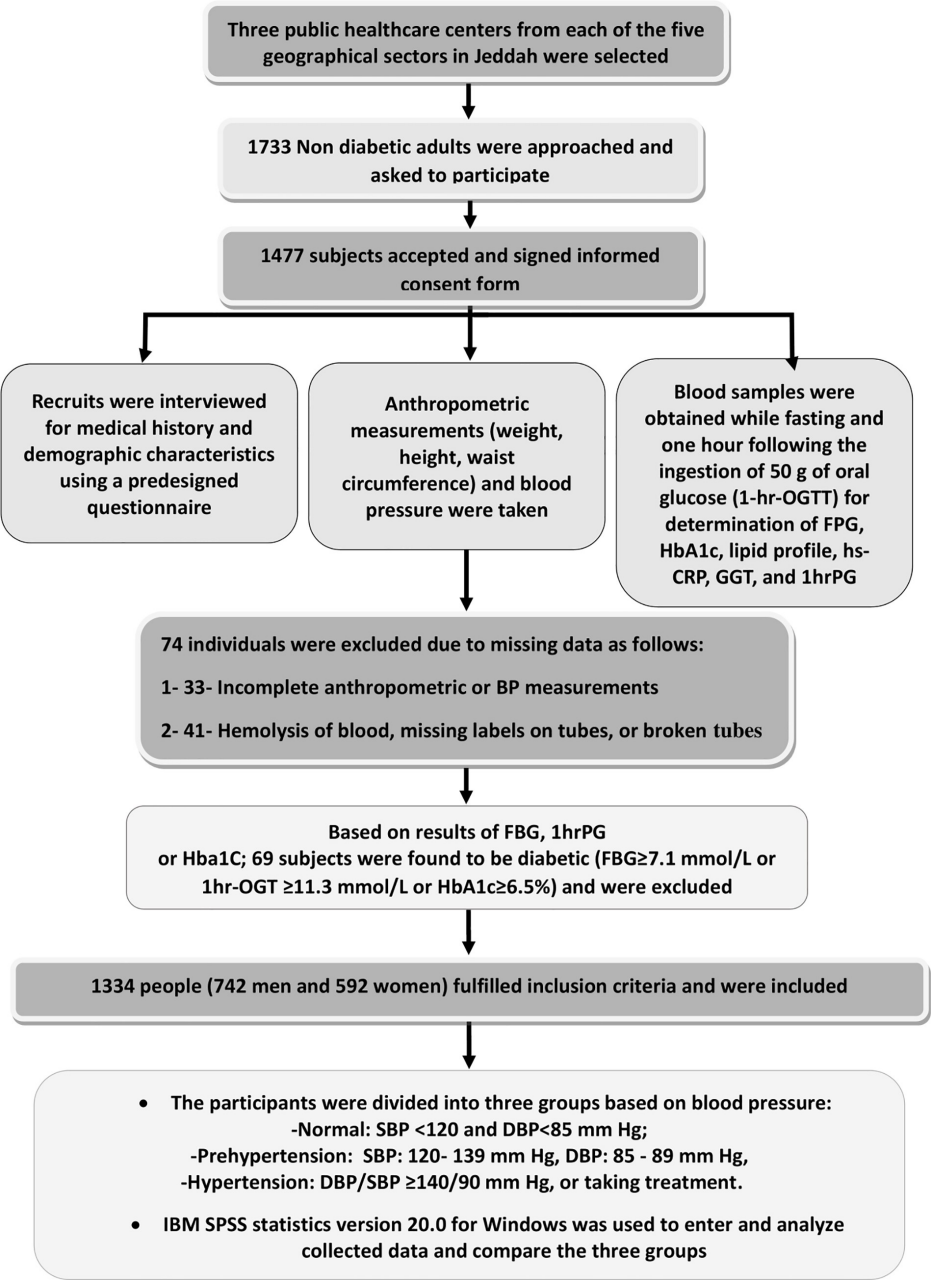
Outline of the different stages of sample collection. A total 37.7% of men and 61% of women had normal blood pressure. Elevated BP was mainly due to increased SBP in both sexes.

A total of 1733 adults were approached, but only 1477 adults accepted to participate and were originally recruited. Individuals were excluded when laboratory samples were missing for various reasons (hemolysis, missing labels, or broken tubes). After laboratory analysis of collected blood samples, and excluding those discovered to have previously undiagnosed diabetes, as well as excluding cases with missing data, complete data for the present analysis were available for 1334 individuals, 742 men and 592 women. Due to the exclusion criteria, very few of the included people (11 men and 7 women) were >64 years of age. Details of the different stages of sample collection are outlined in (Fig 1).

Demographic, clinical, and biochemical **c**haracteristics of study population are presented in Table 1 for men, and Table 2 for women:

**Table 1:** Demographic, clinical and biochemical characteristics of men by the study groups presented as mean ± standard deviation (SD).

Variable	Non-hypertensive (Mean±SD) (N = 280)	Prehypertensive (Mean±SD) (N = 350)	Hypertensive (Mean±SD) (N = 112)	P-Value
Age (years)	29.2 ± 9.0	29.9 ± 8.8	37.2 ± 14.3^b^	**<0.001**
BMI (kg/m^2^)	25.6 ± 0.3	28.0 ± 5.7^a^	30.4 ± .67^b^	**<0.001**
Waist Circumference (cm) Individuals with high values (WC >94 cm), (n,%)	91.5 ± 13.8 (124), 44.4%	97.7 ± 14.9^a^(204), 59.0%	104.4 ± 16.6^b^(80), 74.1%	**<0.001 <0.001**
SBP(mean ± SD) Individuals with high values (SBP≥ 120 mmHg), (n,%)	110± 6.4	125 ± 5.6^a^ (343), 98.0%	137± 15.3^b^(104), 92.9%	**<0.001 <0.001**
DBP Individuals with high values (DBP≥ 85 mmHg), (n,%)	68 ± 9.10	76 ± 8.0^a^ (50), 14.3%	88 ± 11.7^b^(77), 68.5%	**<0.001** **<0.001**
FPG, mmol/l Individuals with high values (FPG≥6.1mmol/l), (n,%)	4.2± 0.74 (1), 0.4%	4.0 ± 1.21^a^(2), 0.8%	4.6± 0.94^b^(9), 6.9%	**<0.001** **<0.001**
1-h, PG mmol/l Individuals with high values (1-h, PG≥8.6mmol/l), (n,%)	6.3± 1.83 (29),11.2%	5.9 ± 2.34^a^(36),11.4%	7.0 ± 1.91^b^(22), 22.7%	**<0.001** **0.010**
HbA1c, % Individuals with high values (HbA1c≥5.7%), (n,%) mmol/mol Individuals with high values (HbA1c≥39 mmol/mol), (n,%)	5.2± 0.42 (22), 8.2% 33.1 ± 0.453 (21),7.9%	5.2 ± 0.38 (34), 10.2% 33.7 ± 4.14 (28), 8.4%	5.3 ± 0.43^b^(18), 17.8% 34.2 ± 4.75^b^(16), 15.7%	**0.036** **0.030** **0.039** **0.014**
Individuals with IGT with any definition (FBG≥6.1mmol/l or 1-h, PG≥8.6mmol/l or HbA1c≥5.7%) (n,%)	(46), 16.4%	(63), 18.0%	(37), 33.3%	**0.001**
TC (mmol/L)	4.65 ± 0.99	4.86 ± 0.92^a^	4.93 ± 0.90^b^	**0.006**
TG (mmol/L)	1.28 ± 0.96	1.41 ± 1.00	1.74 ± 1.03^b^	**0.000**
HDL- C (mmol/L)	1.26 ± 0.23	1.28 ± 0.26	1.18 ± 0.24^b^	**0.001**
LDL- C (mmol/)	3.15 ± 0.94	3.29 ± 0.82	3.41 ± 0.86^b^	**0.021**
hs-CRP (mg/L)	2.41 ± 3.04	3.22 ± 3.58^a^	3.54 ± 3.95^b^	**0.003**
GGT (IU/L)	28.63 ± 18.04	37.53 ± 32.64^a^	38.83 ± 24.68^b^	**<0.001**

n, number of subjects in subgroup; FPG, fasting plasma glucose; 1-hPG, 1-hour plasma glucose; TC, serum total cholesterol; HDL- C, serum high density lipoprotein cholesterol; LDL, serum low density lipoprotein cholesterol; TG, serum triglycerides; hs-CRP,serum highly sensitive C-reactive protein; GGT, gamma glutamyl transferase; P value of ANOVA test for differences between the three groups

a: P<0.05 between normal and pre-hypertensive groups

b: P<0.05 between normal and hypertensive groups

**Table 2:** Demographic and biochemical characteristics of women by studied groups presented as mean ± standard deviation (SD).

Variable	Non-hypertensive (Mean±SD) (N = 361)	Prehypertensive (Mean±SD) (N = 146)	Hypertensive (Mean±SD) (N = 85)	P-Value
Age (years)	31.4 ± 10.5	33.5 ± 12.3^a^	40.9 ± 14.3^b^	**<0.001**
BMI (kg/m^2^)	26.0 ± 5.6	28.7 ± 6.4^a^	31.1 ± 7.5^b^	**<0.001**
Waist Circumference (cm) Individuals with high values (WC>80 cm), (n,%)	84.3±14.3 (202), 57.7%	91.4 ±16.3^a^ (109), 77.3%	98.0 ±0.162^b^(75), 88.2%	**<0.001 <0.001**
SBP(mean ± SD) Individuals with high values (SBP≥ 120 mmHg), (n,%)	104 ± 8.2	122 ± 4.9^a^ (135), 92.5%	133 ± 18.7^b^(72), 84.7%	**<0.001** **<0.001**
DBP Individuals with high values (DBP≥ 85 mmHg), (n,%)	65 ± 7.9	75 ± 8.8^a^ (22), 15.1%	87 ± 11.7^b^(57), 67.1%	**<0.001** **<0.001**
FPG, mmol/l Individuals with high values (FBG≥6.1mmol/l), (n,%)	4.2 ± 0.66 (6), 1.7%	4.3 ± 0.77 (1), 0.7%	4.4 ± 0.77^b^(5), 5.9%	0.117 **0.019**
1-h PG mmol/l Individuals with high values (1-h, PG≥8.6mmol/l), (n,%)	6.2 ± 1.62 (29), 8.7%	6.5 ± 1.69 (12), 9.0%	6.9 ± 1.82^b^ (16), 21.6%	**0.007** **0.004**
HbA1c, % Individuals with high values (HbA1c≥5.7%), (n,%) Mmol/mol Individuals with high values (HbA1c≥39 Mmol/mol), (n,%)	5.2 ± 0.37 (34), 9.7% 33.6± 4.00 (31), 8.9%	5.3 ± 0.37 (15), 10.9% 34.0 ± 3.95 (13), 9.4%	5.3 ± 0.45 (14), 17.5% 34.3 ± 4.96 (13), 16.3%	0.389 0.135 0.364 0.556
Individuals with IGT with any definition (FBG≥6.1mmol/l or 1-h, PG≥8.6mmol/l or HbA1c≥5.7%) (n,%)	(62), 17.2%	(23), 15.8%	(28), 32.9%^b^	**0.002**
TC (mmol/L)	4.76± 0.93	4.80 ± 0.88	5.13 ± 0.98^b^	**0.004**
TG (mmol/L)	0.96 ± 0.48	1.08 ± 0.63^a^	1.30 ± 0.70^b^	**<0.001**
HDL- C (mmol/L)	1.48 ± 0.28	1.48 ± 0.32	1.45 ± 0.25	0.542
LDL- C (mmol/)	3.09 ± 0.81	3.12 ± 0.83	3.43 ± 0.84^b^	**0.003**
hs-CRP (mg/L)	3.42 ± 3.84	4.02 ±4.40	5.13 ± 5.07^b^	**0.004**
GGT (IU/L)	17.82 ± 18.19	19.08 ± 13.09	21.85 ± 26.42	**0.195**

n, number of subjects in subgroup; FPG, fasting plasma glucose; 1-hPG, 1-hour plasma glucose; TC, serum total cholesterol; HDL- C, serum high density lipoprotein cholesterol; LDL, serum low density lipoprotein cholesterol; TG, serum triglycerides; hs-CRP,serum highly sensitive C-reactive protein; GGT, gamma glutamyl transferase; P value of ANOVA test for differences between the three groups

a: P<0.05 between normal and pre-hypertensive studied groups

b: P<0.05 between normal and hypertensive studied groups

Hypertensive men and women were significantly older than their pre-hypertensive and normotensive counterparts. Moreover, means of BMI, and WC, of both pre-hypertensive, and hypertensive groups of men or women were significantly higher than those of their respective normotensive groups.

The means of all measures of glycemia (FPG, 1-h PG, and HbA1c) were all significantly higher in hypertensive compared with normotensive groups of men. However, only the means of FPG and 1-h PG in hypertensive group of women were significantly higher than those of the normotensive group. The percentages of men or women with IGT were significantly higher in the hypertensive groups compared with their respective normotensive groups.

The means of TC, hs-CRP, and GGT of pre-hypertensive and hypertensive groups of men were significantly higher than the means of normotensive group. Moreover, a higher percentage of elevated GGT (Saudi upper limit for men is 86 UL) [47] was also noted in both the pre-hypertensive (6.2%) and hypertensive (4.5%) compared with normotensive (1.8%) group of men. However, the means of TG, and LDL-C were found to be significantly higher, and that of HDL-C lower only in the group of hypertensive men compare to the corresponding means in the normotensive group.

Different results were obtained for women. Only the means of TC, TG, LDL-C, and hs-CRP in the hypertensive group of women were significantly higher than those corresponding means in the normotensive group. In addition, the mean TG was significantly higher in the pre-hypertensive group compared with that of the normotensive group. Elevated GGT (Saudi upper limit for women> 30 UL) [47] was noted in the normotensive group (8.4%), as well as the pre-hypertensive (7.7%) and hypertensive (10.7%) groups of women.

Following multiple logistic regression, the unadjusted odds ratio (OR) and confidence interval (CI) and those adjusted for age alone, and again for age, BMI and WC for the association between the combined groups of people with higher than acceptable BP (pre-hypertension and hypertension), as well as the hypertensive groups alone with CVD risk covariates in men and women is presented in Tables 3 and 4, respectively:

**Table 3:** Unadjusted, age adjusted, and age, BMI and WC adjusted odds ratio (OR) and confidence interval (CI) for CVD risk covariates associated with pre-hypertension and hypertension combined in men and women.

Covariate	Men			Women		
	Unadjusted OR, CI 95%, P-Value	Age adjusted OR, CI 95%, P-Value	Age, BMI and WC adjusted OR, CI 95%, P-Value	Unadjusted OR, CI 95%, P-Value	Age adjusted OR, CI 95%, P-Value	Age, BMI and WC adjusted OR, CI 95%, P-Value
**Elevated FPG, mmol/l** **(FBG≥6.1mmol/l),**	6.81(0.87–52.99),0.067	5.41(0.69–42.6),0.106	4.25(0.54–33.8),0.171	1.58(0.50–4.95),0.435	0.96(0.29–3.17),0.950	0.91(0.28–3.04),0.883
**Elevated 1-h PG mmol/l** **(1-h, PG≥8.6mmol/l),**	1.27(0.79–2.06),0.313	1.09(0.66–1.78),0.746	0.96(0.58–1.60),0.879	1.63(0.94–2.84),0.081	1.22(0.69–2.18),0.493	1.16(0.64–2.13),0.626
**Elevated HbA1c, %** **(HbA1c≥5.7%),** **(HbA1c≥39 Mmol/mol),**	1.51(0.89–2.55),0.124 1.32(0.77–2.27),0.319	1.28(0.74–2.20),0.380 1.09(0.62–1.92),0.766	1.07(0.61–1.88),0.816 0.89(0.49–1.60),0.686	1.43(0.84–2.42),0.187 1.39(0.80–2.42),0.238	1.04(0.60–1.82),0.886 1.00(0.56–1.79),0.994	0.94(0.52–1.68),0.823 0.89(0.48–1.65),0.713
**Elevated TC, mmol/l** **TC≥5.18mmol/l**	1.45(1.05–1.99),**0.024**	1.33(0.96–1.84),0.089	1.23(0.88–1.72),0.230	1.35(0.95–1.92),0.096	1.01(0.69–1.47),0.978	0.89(0.60–1.33),0.576
**Elevated TG, mmol/l** **TG≥1.70mmol/l**	1.95(1.36–2.81),**0.000**	1.74(1.20–2.53),**0.004**	1.52(1.03–2.24),**0.035**	1.97(1.21–3.21),**0.006**	1.45(0.87–2.42),0.159	1.21(0.71–2.08),0.490
**Low HDL- C level, mmol/l** **HDL-C<1.04 M mmol/l, <1.3F mmol/l**	1.45(0.99–2.12),0.055	1.24(0.84–1.84),0.279	1.14(0.75–1.71),0.542	1.32(0.92–1.92)0.136	1.22(0.83–1.77),0.312	1.04(0.70–1.55),0.851
**Elevated LDL- C, mmol/l** **LDL-C≥3.37 mmol/l**	1.31(0.97–1.78),0.081	1.23(0.90–1.67),0.198	1.12(0.81–1.54),0.503	1.56(1.11–2.21),**0.011**	1.30(0.91–1.87),0.198	1.11(0.76–1.62),0.590
**Elevated hs-CRP, mg/l** **hs-CRP>3mg/l**	1.81(1.29–2.55),**0.001**	1.81(1.28–2.55),**0.001**	1.30(0.90–1.87),0.168	1.45(1.03–2.06),**0.035**	1.16(0.81–1.68),0.423	0.70(0.45–1.07),0.100
**Elevated GGT, U/l** **GGT>86 U/l M, >30U/l F**	3.28(1.24–8.64),**0.016**	3.25(1.23–8.58),**0.018**	2.60(0.96–6.98),0.059	1.06(0.59–1.91),0.851	0.80(0.43–1.49),0.486	0.68(0.35–1.29),0.238

**Table 4:** Unadjusted, age adjusted, and age, BMI and WC adjusted odds ratio (OR), and confidence interval (CI) for CVD risk covariates associated with hypertension in men and women.

Covariate	Men			Women		
	Unadjusted OR, CI 95%, P-Value	Age adjusted OR, CI 95%, P-Value	Age, BMI and WC adjusted OR, CI 95%, P-Value	Unadjusted OR, CI 95%, P-Value	Age adjusted OR, CI 95%, P-Value	Age, BMI and WC adjusted OR, CI 95%, P-Value
**Elevated FPG, mmol/l** **(FBG≥6.1mmol/l),**	18.26(4.86–68.58),**0.000**	12.81(3.26–50.32),**0.000**	10.98(2.75–43.79),**0.001**	4.46(1.38–14.41),**0.012**	2.22(0.63–7.79),0.212	2.19(0.62–7.71),0.224
**Elevated 1-h PG mmol/l** **(1-h, PG≥8.6mmol/l),**	2.27(1.32–3.89**),0.003**	1.47(0.82–2.64),0.197	1.34(0.73–2.44),0.346	2.85(1.51–5.41),**0.001**	1.79(0.90–3.57),0.096	1.69(0.85–3.39),0.137
**Elevated HbA1c, %** **(HbA1c≥5.7%),** **(HbA1c≥39 Mmol/mol),**	2.09(1.17–3.72),**0.013** 2.09(1.14–3.84),**0.017**	1.28(0.68–2.41),0.453 1.23(0.63–2.41),0.543	1.09(0.57–2.08),0.799 1.02(0.513–2.02),0.965	1. 90(0.99–3.63),**0.052** 1.96(1.00–3.82),**0.049**	1.12(0.56–2.26),0.747 1.14(0.55–2.35),0.720	1.08(0.53–2.19),0.835 1.09(0.52–2.27),0.817
**Elevated TC, mmol/l** **TC≥5.18mmol/l**	1.90(1.27–2.86),**0.002**	1.60(1.05–2.45),**0.030**	1.60(1.03–2.49),**0.036**	2.77(1.73–4.42),**0.000**	1.87(1.13–3.08),**0.015**	1.78(1.07–2.97),**0.027**
**Elevated TG, mmol/l** **TG≥1.70mmol/l**	2.51(1.65–3.81),**0.000**	1.94(1.25–3.01),**0.003**	1.72(1.09–2.71),**0.021**	3.48(2.00–6.05),**0.000**	2.23(1.24–4.03),**0.008**	2.03(1.12–3.70),**0.020**
**Low HDL- C level, mmol/l** **HDL-C<1.04 M mmol/l, <1.3F mmol/l**	2.70(1.75–4.18),**0.000**	1.89(1.18–3.02),**0.008**	1.77(1.09–2.88),**0.021**	1.66(1.02–2.70)**0.041**	1.46(0.88–2.43),0.139	1.27(0.76–2.14),0.366
**Elevated LDL- C, mmol/l** **LDL-C≥3.37 mmol/l**	1.85(1.23–2.77),**0.003**	1.62(1.06–2.47)**0.025**	1.59(1.02–2.47),**0.039**	2.21(1.39–3.53),**0.001**	1.67(1.02–2.73),**0.040**	1.50 (0.91–2.48),0.112
**Elevated hs-CRP, mg/l** **hs-CRP>3mg/l**	1.32(0.86–2.02),0.207	1.29(0.82–2.02),0.272	0.89(0.54–1.46),0.648	1.52(0.94–2.46),0.091	1.00(0.60–1.69),0.992	0.64(0.35–1.17),0.148
**Elevated GGT, U/l** **GGT>86 U/l M, >30U/l F**	1.06(0.40–2.82),0.909	1.09(0.40–2.94),0.872	0.83(0.29–2.36),0.723	1.34(0.63–2.87),0.451	0.90(0.39–1.97),0.754	0.76(0.34–1.72),0.509

After adjusting for age, elevated TG, hs-CRP, and GGT remained significantly associated with higher than optimal blood pressure in men, but no association was noted in women. After adjusting for age, BMI, and WC, only elevated TG remained significantly associated with higher than optimal blood pressure in men, while no significant associations remained in women with any of the measured variables (Table 3).

After adjusting for age only, elevated FPG, TC, TG, LDL-C, and low HDL- C remained significantly associated with hypertension in men, but only elevated TC, TG and LDL-C maintained association in women. After adjusting for age, BMI, and WC, elevated FPG, TC, TG and LDL- C, as well as low HDL-C remained significantly associated with hypertension in men, while in women an association was found only for elevated TG and TC (Table 4)

## Discussion

Increased risks of CVD and mortality are associated with higher than optimal blood pressure [7–10]. The risk has been reported to be further increased when combined with diabetes or pre-diabetes in Chinese population [48]. The presence of other risk factors for CVD, such as chronic inflammation, and dyslipidemia, in addition to increased blood pressure might raise the risk of the disease further [49]. However, there are differences in relative weight of CVD risk factors between ethnic groups [50], and there are no such studies conducted on Saudi population in spite of the high prevalence of pre-hypertension and hypertension as reported earlier [51–53], and as noted also in this study, as well as the high prevalence of diabetes and pre-diabetes among the Saudis [54]. Therefore, this study was conducted to investigate an important aspect related to improvement in healthcare, namely the association between higher than optimal blood pressure and other known laboratory measured risk factors of CVD in our population, in the hope of formulating guideline for necessary basic laboratory tests that should be requested by practitioners treating non diabetic patients with higher than optimal blood pressure.

A high percentage of our study population had elevated blood pressure, with only 37.7% of men, and 61% of women having normal values, and with pre-hypertension being present in 47.2% of men, and 24.7% women, while 15.1% of men and 14.6% of women and were hypertensive. In the latest Saudi national survey conducted in 2013 [52], 15.2% and 40.6% of Saudis aged 15 years or older were hypertensive or borderline hypertensive, which is close to our findings. Furthermore, similar to our findings, the same survey reported higher risk of hypertension among men compared to women [52]. In addition, a lower prevalence of hypertension and pre-hypertension among women compared to men was also reported in a more recent Saudi study [51]. This is in agreement with reports from studies in other countries in the region [55], as well as older studies on populations from different parts of the globe [56].

Aging is well-known to be associated with increased risk of hypertension in various populations [57–59], and also noted in previous Saudi studies [51–53]. In concordance with this, the mean age of people with hypertension in our study was significantly higher than the mean age of normotensive people. In addition, the means of BMI and WC in the hypertensive and pre-hypertensive people were significantly higher than the corresponding means of those with normal BP. Overweight and obesity, especially when accompanied with abdominal obesity, are major causes of hypertension [60–62]. In previous Saudi studies, higher than normal weight was reported to be associated with increased prevalence [53] and increased risk of hypertension [51, 52]. However, these studies had not investigated sex differences in risk assessment, nor did they adjust for age when reporting association. In an earlier survey conducted in Jeddah by our group, obesity and overweight were found to be associated with an increased risk of hypertension in men but not in women after adjusting for age [36]. Therefore, in this study while investigating the association between increased blood pressure and laboratory measured CVD risk factors, age alone, as well as age, BMI and WC were adjusted for to account for their possible influence on results.

After adjustment for age, elevated TG, hs-cRP, and GGT remained significantly associated with higher than optimal blood pressure (pre-hypertension and hypertension combined) in men, but no association was noted in women. However, following similar adjustment, elevated FPG, TC, TG, LDL-C, and low HDL-C remained significantly associated with hypertension in men, but only elevated TC, TG and LDL-C maintained association in women.

After adjustment for age, BMI and WC, above than optimal blood pressure (pre-hypertension and hypertension combined) was associated only with increased level of serum TG in men but not women. However, following similar adjustments, hypertension was significantly associated with elevated FPG, TC, TG, and LDL-C, as well as low HDL-C in men, while significant association was found only for elevated TG, and TC in women. These results emphasize gender differences in our population, and should be taken into account when considering management.

However, no significant association was found between high blood pressure and hs-CRP or GTT, even though both are considered as markers of inflammation and are associated with increased risk of CVD. Indeed, serum CRP level is reported to be a powerful predictor of ischemic CVD events in apparently healthy subjects, as well as in patients with stable or unstable angina [37, 38]. Furthermore, CRP and blood pressure are considered to be independent determinants of CVD risk, increasing the risk when combined [63, 64]. In addition, the same group of researchers, as well as others reported that CRP level was positively associated with systolic blood pressure, and hypertension [63, 64]. However, another study contradicted this and found that elevated CRP levels were not associated with, and did not appear to lead to elevated blood pressure, after adjustment for various confounders [65], which substantiates our results when adjustment was made for age, BMI and WC.

GGT has been reported to be involved in the pathogenesis of hypertension in a Chinese three-year follow-up study on normotensive and hypertensive subjects [66], while another Chinese study reported that GGT showed strong positive correlations with systolic blood pressure and diastolic blood pressure [67]. In addition, a Korean study reported that high serum GGT levels, and even high normal level, were associated with a higher risk of incident hypertension, particularly in alcohol drinkers and non-overweight individuals [68]. Moreover, young patients with pre-hypertension were reported to have higher serum GGT levels compared with healthy subjects in a small case-control study in Turkey [69]. In our study, elevated levels of GGT was noted also among normotensive women, in addition to women with high blood pressure, while pre-hypertensive and hypertensive men had a significantly higher mean level of GGT compared with normotensive men. Additionally, in this study an association with higher than optimal blood pressure was found after adjustment for age only in men, but not women. Yet, after adjusting for age, BMI and WC no significant association was found between GGT and higher than optimal blood pressure. A recent meta-analysis evaluating association between development of hypertension and GGT level found that the reported direct association between GGT level and risk of hypertension was more significant in Asians and male subgroups compared to non-Asians and female subgroups, explaining this by the fact that Asians have more prevalence of hepatic diseases, which was not accounted for in these studies, and that men drink more alcoholic beverages than women in the studied populations [70]. In addition, when they performed subgroup analysis in non-drinkers, only a non-statistically significant increased trend was found, which is comparable to our results since our study participants were non-drinkers.

The Saudi Hypertension Guidelines 2018 [71] recommended the measurement of complete lipid profile, and fasting blood glucose in all diagnosed hypertensive patients as basic investigations to improve management and decrease risk of CVD. This is in keeping with the European [72] and American [46] guidelines. However, these guidelines were not endorsed by the "Saudi Ministry of Health", and hence, have not been adhered to strictly, leaving the matter to the interest and judgment of the treating physician. Furthermore, there are no specific Saudi guidelines for basic investigations in the case of pre-hypertension, which means that no specific laboratory investigations are usually requested. As mentioned earlier, the risk of CVD is very much increased when combined with diabetes or pre-diabetes [48], especially with elevated post-challenge glucose. Indeed, the co-existence of multiple CVD risk factors in any person results in an effect that is greater than the sum of the effects of its individual components [72], leading to a shift towards higher risk stratification and necessitating different management strategy to prevent, or at least delay, the development of CVD, which contributes to 31% of all deaths globally [73], and to 45% of all deaths in Saudi Arabia (Al Habib, 2020) [74] mainly due to poor preventive management strategies. Therefore, this study provides important, evidence based information, much needed in our region to improve measures for the prevention of CVD by guiding treating physicians to the most likely required set of tests for risk stratification, and hence improved management.

Our study has a few limitations. The first one is common to all cross-sectional studies where only an association between studied variables, but not causation, can be inferred. Another limitation is that only few people were included in the older age group (>64 years) due to being already diagnosed with diabetes one of the conditions in the exclusion criteria. Strengths of our study include the involvement of well-trained data collectors and the use of well-standardized methods for data collection. Secondly, to avoid bias, PHCCs and participants were selected randomly. Moreover, accuracy of laboratory results was assured by conducting all measurements in one accredited laboratory.

In conclusion, based on our results, hypertensive men and women, and pre-hypertensive men are more likely to have dyslipidemia, increasing their risk for CVDs. Furthermore, hypertensive men are more likely to have impaired fasting glucose with a further increase in risk. Therefore, it is highly recommended that all physicians include the estimation of lipid profile and fasting plasma glucose in their routinely requested tests for all diagnosed hypertensive patients. In addition, the estimation of fasting serum TG at least, if not lipid profile, should be requested for all pre-hypertensive men. Failure to do so is expected to lead to less than optimal management of elevated blood pressure needed for the prevention of CVD.

## Supporting information

S1 Data (XLSX)
